# Micro-Plasticity of Genomes As Illustrated by the Evolution of Glutathione Transferases in 12 *Drosophila* Species

**DOI:** 10.1371/journal.pone.0109518

**Published:** 2014-10-13

**Authors:** Chonticha Saisawang, Albert J. Ketterman

**Affiliations:** Institute of Molecular Biosciences, Mahidol University, Salaya Campus, Nakhon Pathom, Thailand; Utah State University, United States of America

## Abstract

Glutathione transferases (GST) are an ancient superfamily comprising a large number of paralogous proteins in a single organism. This multiplicity of GSTs has allowed the copies to diverge for neofunctionalization with proposed roles ranging from detoxication and oxidative stress response to involvement in signal transduction cascades. We performed a comparative genomic analysis using FlyBase annotations and *Drosophila melanogaster* GST sequences as templates to further annotate the GST orthologs in the 12 *Drosophila* sequenced genomes. We found that GST genes in the *Drosophila* subgenera have undergone repeated local duplications followed by transposition, inversion, and micro-rearrangements of these copies. The colinearity and orientations of the orthologous GST genes appear to be unique in many of the species which suggests that genomic rearrangement events have occurred multiple times during speciation. The high micro-plasticity of the genomes appears to have a functional contribution utilized for evolution of this gene family.

## Introduction

*Drosophila melanogaster* is one of the best known dipteran, or ‘true fly’, and has been extensively studied as a model organism for more than a century [1]. The release of the completed *D. melanogaster* genome sequence in 2000 signaled the beginning of the genomic era [2]. In the last decade a further 11 *Drosophila* species have been sequenced. The genomic data from these 12 closely related species has facilitated comparative studies which show many coding sequences have been preserved during species divergence [3]–[6].

In this investigation, we focus on the cytosolic glutathione transferase (GST) superfamily in the 12 *Drosophila* species. Comparative genomics is a powerful tool which has allowed us to identify the GST orthologous protein encoding genes in the 12 diverse species. To identify the GST orthologs, the sequences of *D. melanogaster* were used as reference templates. GSTs are well-known detoxification enzymes which catalyze the reaction of glutathione conjugation to various toxic electrophilic compounds [7]. GSTs are an ancient superfamily comprising a large number of paralogous proteins in a single organism. The soluble cytosolic GSTs constitute the largest GST family in addition to the mitochondrial GSTs [8], [9] and the membrane-associated proteins involved in eicosanoid and glutathione metabolism (MAPEG) [10], [11]. The genes of the cytosolic GST superfamily are organized in multiple clusters on different chromosomes. The genes of GSTs from the same class tend to be clustered together. This is most likely due to the multiple genes originating from tandem gene duplication events. GST isoforms are grouped into different classes according to sequence identity and similarity of their final protein products [7]. There are at least 7 GST classes in mammals (Alpha, Mu, Pi, Omega, Theta, Sigma and Zeta), 7 classes in plants (Tau, Phi, Lambda, Zeta, Theta, DHA and EF1Bγ) [12], [13] and 6 classes in insects (Delta, Epsilon, Omega, Theta, Sigma and Zeta) [14]. This existence of numerous GST isoforms is believed to contribute to the enzymatic responses for cell protection from many different endogenous and exogenous toxic compounds. As previously reported, the GST family members recognize at least 100 different xenobiotic substrates [15]. Moreover this multiplicity of GSTs has allowed the enzymes to diverge and assume non-detoxification functions. For example, the GST Omega class has been shown to be associated with Alzheimer and Parkinson's disease [16]; whereas GST Pi, Mu and Alpha classes in human were shown to interact and regulate several steps in MAP kinase signal cascades [17]–[23].

**Figure 1:**
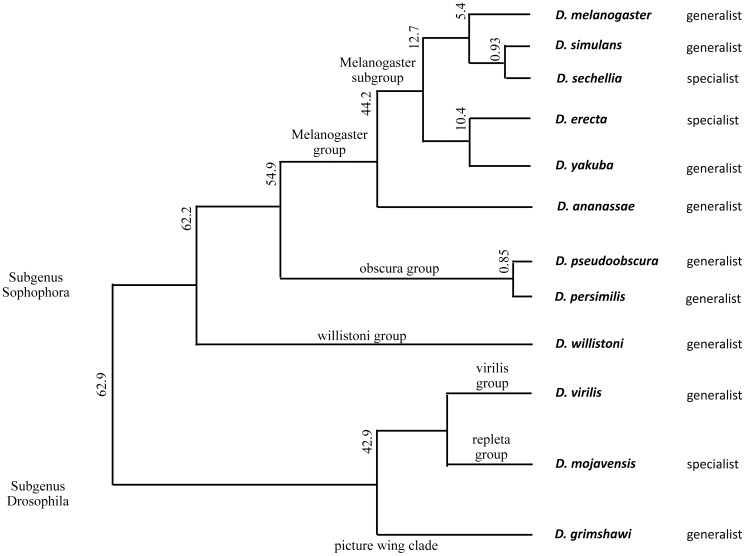
Phylogenetic relationships of the 12 *Drosophila* species. The numbers beside the branches are estimated divergence times in millions of years [32]. The generalist and specialist tags refer to the species feeding habits: *D. melanogaster* [34], *D. simulans* [34], *D. sechellia* [34], *D. erecta* [34], *D. yakuba* [24], *D. ananassae* [34], *D. pseudoobscura* [24], *D. persimilis* [24], *D. willistoni* [24], *D. virilis* [24], *D. mojavensis* [24] and *D. grimshawi* [35].

As stated above, the coding sequences that have been previously annotated in *D. melanogaster* were used as sequence templates to identify the orthologous GST protein encoding genes in the other 11 sister species. The eight sister species of *D. melanogaster*, in subgenus Sophophora, are *D. simulans*, *D. sechellia*, *D. yakuba*, *D. erecta*, *D. ananassae*, *D. pseudoobscura*, *D. persimilis* and *D. willistoni* (Figure 1). The first four species mentioned are the most closely related to *D. melanogaster* and belong to the melanogaster subgroup. The subgenus *Drosophila* contains *D. mojavensis*, *D. virilis* and *D. grimshawi*, and are the most distantly related species to *D. melanogaster* with about 60 million years of divergence time [24]. Although a small number of orthologous GSTs have been studied in different *Drosophila* species [25]–[27], the present study encompasses the whole cytosolic GST superfamily in the 12 species. It should be emphasized that although great innovations in gene prediction have been made using computer program analysis, the accuracy in gene annotation is far from complete, necessitating manual curation. The quality of the genome sequences was of some concern as *D. simulans*, *D. sechellia* and *D. persimilis* were sequenced to a low coverage of only about 2–4 times of the total genome size; whereas, the other species were sequenced to greater than 8 times coverage [4], [28]. To increase the accuracy in the GST gene annotation, we have made a comprehensive sequence analysis augmented with manual curation. The analysis of these *Drosophila* genomes also has yielded some insight into the *Drosophila* GST family evolution as well as observations of several interesting features of the *Drosophila* genomes which are discussed below.

## Results

In the *D. melanogaster* genome, thirty-six genes encode a total of 41 cytosolic GST proteins (Table 1) [14]. These have been named based on the unified GST nomenclature and categorized into 6 different classes [14]. However, many of the GSTs in the other 11 *Drosophila* species still lack this categorization. Therefore we have manually analyzed each gene by comparing the sequences from the 11 *Drosophila* species to a *D. melanogaster* template. Although the nucleotide sequences of the *Drosophila* species reveal codon usage bias, the codon preferences of the amino acids is sufficient for identifying the GST proteins. Due to a clear orthology for the 11 species with *D. melanogaster*, we were able to name and classify the GSTs into the six classes found in insects; Delta (D), Epsilon (E), Omega (O), Theta (T), Zeta (Z) and Sigma (S). Many orthologs have been previously annotated by FlyBase, for example, GSTD1 orthologs in the 4 species; *D. simulans*, *D. sechellia, D. erecta* and *D. yakuba*, as well as GSTE2 and GSTE3 of *D. yakuba*. However the number of genes with orthology to a single *D. melanogaster* gene in each species is varied which is a problem for GST annotation (Table S1 in file S1). In addition, the genomic comparison also showed several inaccuracies in the gene annotations, such as wrong translation initiation sites, wrong exon-intron boundaries and no identified alternatively spliced genes. The identified ambiguities, annotation errors, sequencing errors, pseudogenes and alternative splicing are presented below.

**Table 1:** Glutathione transferases in the 12 *Drosophila* species.

*Drosophila* species	Number of genes	Number of Proteins
*D. melanogaster*	36	41
*D. simulans*	36	41
*D. sechellia*	36	39
*D. erecta*	37	42
*D. yakuba*	39	43
*D. ananassae*	43	47
*D. persimilis*	32	35
*D. pseudoobscura*	31	36
*D. willistoni*	40	43
*D. virillis*	30	32
*D. mojavensis*	28	32
*D. grimshawi*	27	29

The numbers of genes and proteins transcribed are from FlyBase annotations and the present curation. The present curation numbers include proposed corrections for genes and protein expressions.

### Gene annotation errors

Using *D. melanogaster* as a model species, we identified several errors in the annotations for GSTs in the other species. Seven GSTs from 5 *Drosophila* species have been annotated to be either too long or too short compared to the size of the *D. melanogaster* GST genes. The usual length of GST encoding sequence is from 600 to 800 bp. Several short sequences do not contain the critical conserved serine/tyrosine residue in the active site [29]. These key residues also appear to play roles in folding, stability as well as catalyzing the conjugation of the GSH substrate [30]. We believe that these annotation errors result from automatic algorithm predictions employed to process the large genome data sets. We wish to propose the following GST annotations based on the *D. melanogaster* annotation and our manual curation.

1. *Dsim\GD17126* (*DsimGSTT4*) is a *D. melanogaster* GSTT4 ortholog. Its gene size reported by FlyBase is 864 bp. A comparison of the nucleotide sequences of *DsimGSTT4* and *DmelGSTT4*, show that the annotated exons 2 and 3 of *DsimGSTT4* are actually part of an intron in the *DmelGSTT4* gene (Figure S1 in file S1). These 2 extra exons make *DsimGSTT4* larger than other GSTT4 orthologs. If the 2 exons are presumed to be intron, the size of *DsimGSTT4* would be 714 bp and show 100% amino acid sequence identity to DmelGSTT4.
2. *Dere\GG21888* (*DereGSTE6*) is a *D. melanogaster* GSTE6 ortholog. Its gene size reported by FlyBase is 723 bp. It is larger than other GSTE6 orthologs because of an incorrectly annotated ATG start site. Using *DmelGSTE6* as template, the correct ATG start site should be the next ATG after the annotated one which would reduce its size to 669 bp and show 93% amino acid sequence identity to DmelGSTE6 (Figure S2 in file S1).
3. *Dana\GF17942* is a member of Delta class. The gene reported by FlyBase is 1197 bp with 2 exons. Again we have found that a more likely ATG start site is at the end of the annotated intron 1. This sequence codes for the first 5 amino acids of the protein and is contiguous with the second exon (Figure S3 in file S1). Thus *Dana\GF17942* would be one single exon of 663 bp. This proposed sequence would translate to give 64% amino acid sequence identity to DmelGSTD2.
4. *Dana\GF11968* (*DanaGSTE11*) is a *D. melanogaster* GSTE11 ortholog. Its gene size reported by FlyBase is 2919 bp. This is a similar case to the above mentioned annotation with proposed exon in annotated intron. In this case the proposed last 45 nucleotides of the coding region are located in the second intron (Figure S4 in file S1). This sequence includes the stop codon so a read-through from exon 2 including these 45 nucleotides would yield a *DanaGSTE11* that encodes a GST protein of 225 amino acids. This proposed sequence shows 87% amino acid sequence identity to DmelGSTE11.
5. *Dvir\GJ22855* is a Delta class GST. Its gene size reported by FlyBase is 603 bp. From sequence analysis, we found the ATG start site should be 48 nucleotides upstream of the annotated site (Figure S5 in file S1). These 48 nucleotides are in-frame and contiguous with the annotated exon. This longer exon then translates to a GST protein 216 amino acids long with 67% amino acid identity to DmelGSTD2.
6. *Dvir\GJ22856* is located next to *Dvir\GJ22855*. Its annotated size is also 603 bp. We found a more appropriate start site 33 nucleotides upstream of the annotated ATG start site. This longer exon translates to yield a conserved Delta class sequence which results in a protein 211 amino acids long (Figure S6 in file S1). This protein also appears to be an ortholog to DmelGSTD2 with 64% amino acid identity.
7. *Dvir\GJ23571* (*DvirGSTZ1*) is annotated to be an ortholog of GSTZ1 from *D. melanogaster*. Its gene size reported by FlyBase is 489 bp. This gene size would encode a truncated protein. However, we found that the ATG start site should be 255 nucleotides upstream of the annotated one (Figure S7 in file S1). This proposed start site would translate to a GST protein 247 amino acids long with 61% amino acid identity to DmelGSTZ1.

### Sequencing errors

Incomplete genome sequencing also contributes to the errors in sequence annotation. This is especially evident for the genomes of *D. sechellia* and *D. persimilis* that were sequenced to low coverage, 4.9X and 4.1X [28]. In addition, the genome of *D. simulans* which was performed as a mosaic assembly was also sequenced to a low coverage of 2.8X [28]. The annotation of the genome of these species therefore becomes difficult. The incompleteness of genome sequencing has yielded incorrect sequence annotation for 3 GST genes, *Dsec\GM23038* (*DsecGSTT3*), *Dper\GL26999* (*DperGSTT4*) as well as not identifying a proposed alternatively spliced *Dper\GL13668* (*DperGSTZ2*). These annotations are described below.

1. *Dsec\GM23038* (*DsecGSTT3*) is annotated to be a *DmelGSTT3* ortholog which codes for a protein of 87 amino acids. Using this short sequence to align with GSTT3 of *D. melanogaster*, the first 40 and the last 47 amino acids align very well to DmelGSTT3 with a gap in between (Figure S8 in file S1). However upon retrieving its gene region from FlyBase, it was found that the DNA sequencing is incomplete as shown by Ns in the middle of the gene. Therefore we suspect that this is a sequencing error of the original clone and Dsec\GM23038 would be an orthologous protein to DmelGSTT3. Nevertheless, the gene needs to be re-sequenced for confirmation.
2. *Dper\GL26999* (*DperGSTT4*) is annotated to be a *DmelGSTT4* ortholog which codes for a protein of 280 amino acids. This is a similar case as *GSTT3* of *D. sechellia* with the gene incompletely sequenced and shown as N's. Moreover we suggest that the annotated exons 2 and 3 of *D. persimilis* should be part of a large single intron. Therefore the first exon will encode the first 39 amino acid residues and the next 53 amino acid residues are encoded in the incomplete gene region and annotated exon 4 (Figure S9 in file S1). The remainder of the sequence is quite conserved. Presuming the annotated exons 2 and 3 to be intron, the size of DperGSTT4 is reduced 93 amino acids. Thus 39 residues from the first exon and including the missing 53 residues as well as the 148 residues from exons 4, 5 and 6 of the sequence, the size of DperGSTT4 will be approximately 240 amino acids which is comparable to GSTT4 in other species. However; again, the whole gene needs to be re-sequenced, especially the missing part.
3. *Dper\GL13668* is reported to be a *DmelGSTZ2* ortholog. This gene would actually provide alternative transcripts encoding 3 final spliced protein products. The alternative splicing of this will be discussed in a following section. We found incomplete sequence in the intron region of *GSTZ2B* variant which does not affect the protein coding sequence. However the first exon of *GSTZ2A* and *GSTZ2C* are also located in this same intron (Figure S10 in file S1). Therefore we suggest that the GSTZ2A and GSTZ2C variants exist in *D. persimilis* as alternatively spliced products.

### Pseudogenes

A pseudogene is a gene sequence that would translate to a non-functional protein. In the 11 *Drosophila* species, we found at least 9 GST genes suspected to be pseudogenes. These sequences were matched with the orthologous genes in the *D. melanogaster* genome. All of the matched genes tend to be shorter than normal GST sizes which appear to be a consequence of frame shifts or premature stop codons. These 9 GST gene annotations are described below.

1. *Dsec\GM24019* is a GST Delta class gene. Its reported gene sequence is 348 bp long. It aligns very well with *DmelGSTD6* at the 3′end of the gene. Although 69 nucleotides upstream of the annotated ATG start site is conserved sequence of Delta class, this slightly longer protein would still be too short to be a viable GST enzyme as it's missing the first 100 amino acids of the protein which consists of the N-terminus domain containing the G-site or GSH binding site (Figure S11 in file S1).
2. *Dsec\GM21877* is placed into GST Epsilon class since its short sequence (447 bp) aligns well with the 3′end of other Epsilon class members (Figure S12 in file S1). In addition, a BLAST search hits *Dmel\CR43687*, a pseudogene in the middle of the *D. melanogaster* Epsilon GST gene cluster.
3. *Dyak\GE11955* codes for a protein of 163 amino acids and is reported to be a *DmelGSTE1* ortholog. Again, by comparing the gene sequence of this *DyakGSTE1* with *DmelGSTE1*, it was found that part of the intron shows conserved sequence to *DmelGSTE1* (Figure S13 in file S1). However, our reanalyzed gene sequence still does not encode for a full-length GSTE1 and it is also missing the critical conserved active site sequence (which encodes Ser-His-Ala-Ile) of Epsilon class.
4. *Dana\GF17941* is a member of GST Delta class. Its gene sequence is 540 bp which codes for a protein of 179 amino acids in length. Dana\GF17941 shows similar sequence to the C-terminal part of DmelGSTD2, GSTD4 and GSTD5 but it is missing the first 38 amino acids which consist of important G-site residues (Figure S14 in file S1). There is no possible coding sequence upstream of the annotated ATG start site.
5. *Dper\GL17151* appears to be a Theta GST ortholog which codes for a protein of 111 amino acids. It shows high sequence conservation to DmelGSTT2 at the N-terminus (Figure S15 in file S1). Although this gene may translate to protein product, this protein would not function as an active GST enzyme as it is missing 117 amino acids or the C-terminus half of the protein that generates the hydrophobic substrate binding site (H-site).
6. *Dper\GL26929* also appears to be a Theta GST ortholog which codes for a truncated protein of 163 amino acids. Although this gene is not located on the same scaffold as other Theta gene members, its translated product shows the greatest amino acid identity, 27% and 29%, to DmelGSTT1 and T2, respectively (Figure S16 in file S1).
7. *Dwil\GK11203* is a GST Delta class member. The annotated sequence codes for a protein of only 108 amino acids. We have found conserved sequence of Delta class 91 nucleotides upstream of the annotated ATG start site. Moreover there is a possible ATG start site that would encode 17 amino acids of a GST protein (Figure S17 in file S1). This conserved block also shows high identity to *Dwil\GK11871* which is located next to *Dwil\GK11203* on the same chromosome and is further discussed below in the genomic organization section. Nevertheless about 60 amino acids, after the first 17 amino acids, are still missing and these residues constitute a major portion of the active site. Thus if this is not an error from sequencing, *Dwil\GK11203* appears to be a pseudogene.
8. *Dvir\GJ24387* when translated shows the greatest amino acid identity to DmelGSTD10. The gene region of *Dvir\GJ24387* codes for a protein of only 136 amino acids (Figure S18 in file S1).
9. *Dvir\GJ19066* encodes a peptide of 54 amino acids which shows the greatest amino acid sequence identity to DmelGSTD1. Curiously, *Dvir\GJ19066* is located on the same scaffold as the 2 Theta GSTs. Although we found a possible conserved block of sequence upstream of the annotated ATG start site, the nucleotide sequence for the C-terminus of the protein is still missing. Obviously this pseudogene which encodes a ¼ length protein would not be expressing a functional enzyme (Figure S19 in file S1).

### Ambiguous annotation

The mutation rate of different *Drosophila* species by natural selection would influence the gene loss and changes in genome size. Indel mutations are one of the major effectors of genome evolution. From our manual curation of each *Drosophila* GST sequence, we found both gene insertion and deletion mutations in 4 *Drosophila* species. However as previously mentioned, the quality of the genome assembly of *D. sechellia* and *D. simulans* was low. Therefore these findings remain ambiguous as to whether they are errors from genome sequencing or actual indel mutations. These 9 GST gene annotations are described below.

1. *Dsim\GD17492* (*DsimGSTT3*) is annotated to be a *DmelGSTT3* ortholog. This gene would undergo alternative splicing to yield 2 alternatively spliced products. This will be discussed further in the next section. Its gene size reported by FlyBase is 750 bp which codes for smaller GSTT3A and GSTT3B proteins compared to other species. We found that one cytosine (C) appears to be missing (Figure S20 in file S1). Therefore the annotation split exon 5 into 2 exons to keep the remaining DNA sequence in frame. The annotated intron between the split exon 5 and 6 is 56 nucleotides. If the absent cytosine actually is present in the sequence as in *D. melanogaster*, *DsimGSTT3*A and *GSTT3B* will code for proteins of 228 and 268 amino acids and show 98 and 97% amino acid sequence identity to DmelGSTT3A and GSTT3B, respectively.
2. *Dsim\GD11388* (*DsimGSTE11*) is annotated to be a *DmelGSTE11* ortholog and codes for a protein of 251 amino acids. This protein is longer than GSTE11 orthologs in other species. We found a possible ATG start site which codes for the conserved GSTE11 sequence in intron 1 but the end of this intron has a TAG stop codon (Figure S21 in file S1). If the putative stop codon (TAG) at the end of intron 1 is CTG, as in *D. melanogaster*, and the translation start site is from our predicted one, it would encode a protein of 225 amino acid and show 97% sequence identity to DmelGSTE11.
3. *Dsim\GD24922* (*DsimGSTE12*) is annotated to be a *DmelGSTE12* ortholog and codes for a protein of 213 amino acids. This annotated protein is shorter than other GSTE12 orthologs. This is possibly due to one thymidine (T) missing from exon 2 (Figure S22 in file S1). We found that if this absent thymidine is present as it is in *D. melanogaster* and transcription occurs reading through the intron 2 as coding sequence, this would encode a protein of 223 amino acids and show 98% amino acid sequence identity to DmelGSTE12.
4. *Dsec\GM24014* and *Dsec\GM24015* are annotated to be GST protein coding genes. These 2 genes are located next to each other on the same chromosome being only 23 nucleotides apart. These 2 single exons code for proteins of 91 and 115 amino acids, respectively. *Dsec\GM24014* aligns very well with the 5′ end whereas *Dsec\GM24015* aligns with the 3′ end of *DmelGSTD2*. Using the *GSTD2* gene of *D. melanogaster* as template, we found that at nucleotide 261 of the *Dsec\GM24014* exon, one cytidine (C) is missing which results in a stop codon at nucleotide 275 (Figure S23 in file S1). If the missing cytidine is indeed present then merging *Dsec\GM24014* and *Dsec\GM24015*, including the 23 nucleotide gap between the 2 exons, would yield a gene with very high identity to *GSTD2* of *D. melanogaster* (95% and 98% nucleotide and amino acid sequence identity, respectively).
5. *Dsec\GM25062* (*DsecGSTO4*) is annotated to be a *DmelGSTO4* ortholog and codes for a 204 amino acid protein. The gene region of *DsecGSTO4* appears to be completely sequenced. Using *DmelGSTO4* as sequence template, it was found that there are both nucleotide insertions and deletions. There is one cytidine (C) insertion in each of exons 2 and 3 of *DsecGSTO4*, which causes frame shifts so that exon sequence has been annotated as intron. The annotated second intron and part of the third intron align with encoded conserved sequence from exon 2 in *D. melanogaster*. Moreover the second intron of *DsecGSTO4* also is missing one nucleotide. Therefore although we include the second and third introns of *DsecGSTO4* as exon, the reading frame will be shifted which in this case results in a stop codon and incorrect coding protein (Figure S24 in file S1). Previously the DmelGSTO4 has been shown to be PDA (6-acetyl-2-amino-3,7,8,9-tetrahydro-4*H*-pyrimido[4,5-*b*]-[1], [4]diazepin-4-one or pyrimidodiazepine) synthase which converts 2-amino-4-oxo-6-pyruvoyl-5,6,7,8-tetrahydropteridine (also called 6-pyruvoyltetrahydropterin; 6-PTP) into PDA, an important intermediate in eye pigment drosopterin biosynthesis [31]. Another possibility for the sequence ambiguity is that this species sequencing data may actually be from a *sepia* mutant which is the phenotype for mutations of this gene.
6. *Dsec\GM21874* (*DsecGSTE1*) is annotated to be a *DmelGSTE1* ortholog and codes for a protein of 334 amino acids. The annotated exon 1 does not show conserved sequence to any Epsilon class member. In addition, the annotated gene would translate to a protein which is larger than any known GST. We have found a possible ATG start site located in the intron region (34 nucleotides upstream of the annotated second exon (Figure S25 in file S1). However a stop codon results due to an absent thymidine (T) at position 27 from our predicted ATG start site. Nonetheless, if this absent thymine is present as it is in *D. melanogaster*, translation from our predicted site will encode a protein of 206 amino acids. This proposed sequence has 66% amino acid identity to DmelGSTE1. Moreover it will be a single exon as found for GSTE1 of the other *Drosophila* species.
7. *Dsec\GM21098* (*DsecGSTE13*) is annotated to be a *DmelGSTE13* ortholog which codes for a protein of 212 amino acids. This is actually smaller than GSTE13 orthologs in other species due to a one adenosine (A) base insertion which results in a frame shift. It seems that the extra adenosine region was annotated as intron to keep the remaining sequences in-frame (Figure S26 in file S1). If the extra adenosine is absent as in *D. melanogaster*, a read-through of the annotated intron 3 as exon would then yield a protein of 226 amino acids and identity increases to 98% compared to DmelGSTE13.
8. *Dper\GL13668* (*DperGSTZ2*) is annotated to be a *DmelGSTZ2B* ortholog. In addition to the incomplete DNA sequencing as mentioned in the previous Sequencing Error section, there also appears to be an insertion. We found one guanosine (G) insertion in exon 5 which would give a frame shift (Figure S10 in file S1). If the following intron region is included in this exon, to keep the rest of the sequence in frame, this also makes the 3 isoforms of DperGSTZ2 larger than GSTZ2 from the other *Drosophila* species.
9. *Dvir\GJ14962* codes for a protein of 277 amino acids and is identified to be a *DmelGSTE13* ortholog. Again the protein is longer than GSTE13 orthologs in other species. We found one adenosine (A) insertion at nucleotide position 419 of exon 4. This insertion creates a frame shift which reads through its stop codon (TAA) to terminate at the next stop codon (Figure S27 in file S1). If the extra A is absent, as in *D. melanogaster*, the TAA stop codon will be in-frame. The GSTE13 of *D. melanogaster* and *D. virilis* then show 74% amino acid identity to each other.

### Alternative splicing

The FlyBase annotations for the 11 species do not show the alternative splicing found in *D. melanogaster*. In addition, the alternatively spliced products sometimes are reported to originate from different genes. Therefore we have analyzed whether the GST genes in these 11 *Drosophila* species also undergo alternative splicing as in *D. melanogaster*. Our curation reveals that *GSTD11*, *GSTZ2*, *GSTT3* and *GSTO2* in all 11 species would also undergo alternative splicing and GSTO2A and GSTO2B are spliced products from the same gene (Figures S28–S31 in file S1). Exceptions to this are found in *D. mojavensis* and *D. grimshawi*, which have been shown to have only one isoform of *GSTO2* which is the GSTO2B protein. Due to high gene conservation of GSTs throughout the 12 species, we have found a preserved pattern of not only the coding region but also the intron-exon boundaries as well as the untranslated region (UTR) (Figure S32 and Table S2 in file S1).

Although not annotated in FlyBase, the *GSTD11*, *GSTZ2*, *GSTT3* and *GSTO2* in all 11 species would also undergo alternative splicing similar to *D. melanogaster*. As mentioned in a previous section, *DsimGSTT3* and *DperGSTZ2* appear to have indel mutations. However this is somewhat uncertain as these 2 species were sequenced at very low coverage. FlyBase has recently reported a third DmelGSTT3C variant. The coding sequence of DmelGSTT3C is exactly the same as DmelGSTT3A. The difference is the 5′UTR. Since there is no 5′UTR or 3′UTR for the other 11 *Drosophila* species reported yet, the nucleotide sequence that we used in our analysis was the previously annotated *DmelGSTT3A*. In the case of the *DsecGSTT3* gene for which the sequencing is incomplete, we did not include the variant sequences in our analysis.

As mentioned, at present there is no annotation for the 11 *Drosophila* species' untranslated regions. However, based on our manual analysis of each individual gene, we found that both the 5′ and 3′UTR of *GSTZ2* show high sequence conservation to *D. melanogaster*; whereas, the UTR of the *GSTT3* genes are quite varied. *GSTO2* and *GSTD11* of the 4 species; *D. simulans, D. sechellia, D. erecta* and *D. yakuba* reveal highly conserved sequences to the template species especially for the 5′UTR. However the 3′UTR sequences of *GSTD11* are relatively varied.

GSTD11 and GSTZ2 variants show very high amino acid sequence identity to the orthologous *D. melanogaster* genes ranging from 84–100%. GSTD11A and GSTD11B of *D. simulans* show the highest identity whereas *D. grimshawi* show the lowest. The 3 alternatively spliced products of *GSTZ2* show even greater conservation to *D. melanogaster*. The GSTZ2B and GSTZ2C amino acid sequences of *D. simulans, D. sechellia, D. erecta, D. yakuba, D. ananassae, D. persimilis, D. pseudoobscura, D. willistoni* show 100% identity to *D. melanogaster*; although *D. virilis, D. grimshawi and D. mojavensis* have only a single amino acid difference at Val106Ile and Val101Ile of GSTZ2B and GSTZ2C, respectively. GSTD11A and GSTD11B of *D. simulans* also show 100% amino acid sequence identity to *D. melanogaster* (Figure S33 in file S1).

### Genomic organization of the other 11 *Drosophila* species

GSTs comprise a superfamily in *D. melanogaster* as there are at least 36 GST genes in the genome. By using genomic comparison as a tool to identify the GST superfamily members in the other 11 *Drosophila* species, we found different numbers of GST genes in each *Drosophila* species (Table 1 and Table S1 in file S1). Although gene duplication is generally believed to be followed by divergence, the GST genes of all 12 *Drosophila* species seem to be fairly conserved. This actually facilitated the chromosome/scaffold mapping. We have manually analyzed and constructed a genome map for each *Drosophila* species using *D. melanogaster* as a template [14]. Many of the GSTs showed clear orthologs in *D. melanogaster* and FlyBase has annotated or we have curated accordingly. However, some annotated GST genes were fragments or showed orthology to multiple *DmelGSTs* and these cases are specifically discussed below.

#### Drosophila simulans

The genomic organization and distribution of the GST genes on the chromosomes of D. simulans are the most similar to D. melanogaster compared to the other 10 species. D. simulans GSTs show clear orthologous GST isoforms to D. melanogaster; although, the genome sequencing is at low assembly coverage. The D. simulans GST genes are also located on chromosome 3R, 2R, 2L and X (Figure S34A in file S1). The cytological map of D. simulans shows an inverted sequence on chromosome 3R [28]. This paracentric inversion is on the breakpoints 84F1 and 93F6-7. As the GST Delta and Zeta classes are located on this chromosome they are arranged in an inverted orientation, whereas the other 4 GST classes are well clustered and located roughly the same distance from each other when compared to the D. melanogaster template (compare Figure S34A in file S1 and Figure 1 in Reference [14]).

#### Drosophila sechellia

In addition to D. simulans, D. sechellia has also been sequenced to low level coverage which results in errors in scaffold assemblage. Scaffold 0 was reported to be a chimera between chromosome 3R and 3L [28]. As a consequence GST Delta, Zeta and Omega classes were found to be located on the same scaffold. It also shows paracentric inversion for D. sechellia similar to D. simulans. As only Delta and Zeta classes align in the inverted orientation the paracentric inversion appears to occur just in part of chromosome 3R. A BLAST search with Dsec\GM24018 retrieved GSTD2, GSTD4 and GSTD5 of D. melanogaster with 73%, 98% and 70% amino acid identity, respectively. Interestingly D. sechellia also appears to have another GST gene that is a GSTD2 ortholog (as discussed in the Ambiguous annotation section). A BLAST search of Dsec\GM21880 amino acid sequence hits 4 Dmel Epsilon class members, GSTE5 (96% identity), GSTE6 (74% identity), GSTE7 (68% identity) and GSTE8 (72% identity). Dsec\GM21875 is an obvious DmelGSTE2 ortholog with 95% amino acid identity. Although a search with Dsec\GM21878 shows it to be an Epsilon class GST, its highest amino acid sequence identity also is to GSTE2, but with only 64% identity. Two Theta class GSTs, GSTT3 and GSTT4, of D. sechellia are located on different scaffolds, 8 and 21 respectively. Assemblage shows them to be both on the Muller-A element (Dmel chromosome arm X) and in D. melanogaster DmelGSTT3 and GSTT4 are located on chromosome X. Moreover we found incomplete sequencing for DsecGSTT3. We suspect that if the sequencing was complete, Dsec\GM23038 would be an orthologous protein to DmelGSTT3. However the whole gene needs to be sequenced to obtain the missing part of the gene (Figure S34B in file S1).

#### Drosophila erecta

D. erecta possesses both paracentric and pericentric inversions as none of the D. melanogaster GSTs are located on chromosome 2L, whereas GSTT1, GSTT2 and GSTE13 of D. erecta are found on scaffold 4929 which is equivalent to chromosome 2L. This phenomenon comes from a pericentric inversion leading to the fusion of the base of chromosome 2R and 2L near the centromere [28]. Moreover a paracentric inversion also has occurred as these 3 GSTs are oriented in the reverse direction compared to D. melanogaster [28]. The remaining Epsilon members and the Sigma class GST are found on scaffold 4845 (chromosome 2R). Dere\GG21880 and Dere\GG21882 are annotated to be GSTE1 (91% identity) and GSTE2 (90% identity) D. melanogaster orthologs, respectively. However Dere\GG21881 and Dere\GG21885 also are annotated to be GSTE1 and GSTE2 with 79% and 66% amino acid sequence identity, respectively. This is considered to be a very high identity to GSTE1 and GSTE2, as Dere\GG21881 has 26%-51% and Dere\GG21885 has 25%-54% identity to other Epsilon class members. This suggests that these two genes would generate additional GSTE1 and GSTE2 isoform variants in this species. Dere\GG17139 is annotated to be an ortholog to DmelGSTD10 as it shows 91% amino acid identity, although FlyBase also reports Dere\GG17138 to be a DmelGSTD10 ortholog with 95% identity. A sequence search with Dere\GG18783 hit GSTD2 as well as GSTD4 and GSTD5 of D. melanogaster with 79%, 72% and 87% amino acid identity respectively. There is no orthologous protein to D. melanogaster GSTD6 found in D. erecta. As in the two species above, Delta and Zeta class genes show paracentric inversions compared to D. melanogaster as shown in Figure S34C in file S1.

#### Drosophila yakuba

In a similar situation to D. erecta, D. yakuba shows pericentric inversion compared to D. melanogaster, which places Dyak\GE19294 and Dyak\GE19295 on chromosome 2L [28]. The amino acid sequences of these two GSTs hit both GSTT1 and GSTT2 of D. melanogaster. Dyak\GE19294 gives 94% amino acid identity to DmelGSTT1 and 62% to DmelGSTT2; whereas Dyak\GE19295 gives 66% identity to DmelGSTT1 and 92% to DmelGSTT2. Therefore, we annotate these proteins, Dyak\GE19294 and Dyak\GE19295 as DmelGSTT1 and GSTT2 orthologs, respectively. The Epsilon and Sigma classes are still located on chromosome 2R although the chromosome has undergone paracentric inversion. The Omega class gene cluster also has undergone a paracentric inversion on chromosome 3L. Although GSTE2 (90% amino acid identity) and GSTE3 (92% amino acid identity) of D. yakuba have been annotated, Dyak\GE11960 and Dyak\GE11959 also show high identities, 67% and 63% amino acid sequence identity to D. melanogaster GSTE2 and GSTE3, respectively. This indicates the existence of additional isoforms of GSTE2 and GSTE3 in this species. FlyBase has annotated the remaining Epsilon GSTs to their respective orthologs in D. melanogaster. BLAST searching Dyak\GE26173 sequence hit GSTD2, GSTD4 and GSTD5 in D. melanogaster with 82%, 73% and 75% amino acid identity respectively, which yields another ortholog to DmelGSTD2 in D. yakuba (Figure S34D in file S1).

#### Drosophila ananassae

A pericentric inversion also appears to have occurred in this Drosophila species. The inversion changes chromosome X into a metacentric instead of an acrocentric chromosome [28]. D. ananassae GSTT3 and GSTT4 are located on different scaffolds of chromosome X. FlyBase shows Dana\GF17941, Dana\GF17942 and Dana\GF17943 as orthologs of GSTD2, GSTD4 and GSTD5 of D. melanogaster although Dana\GF17941 would appear to be a pseudogene. Curiously, protein product of Dana\GF17943 has greater amino acid identity (73%, 69%, 72%) than Dana\GF17942 (64%, 60%, 61%) to all 3 Delta GST enzymes. Likewise, both Dana\GF17945 and Dana\GF17946 are shown to be orthologs of DmelGSTD7 with a high conservation of 73% and 78% amino acid sequence identity, respectively and 93% identity compared to each other. DmelGSTT1 and GSTT2 have amino acid sequence identities with 3 D. ananassae GSTs, Dana\GF12484 (85%, 66%), Dana\GF12485 (63%, 71%) and Dana\GF12486 (72%, 74%), respectively. Dana\GF12484 shows the highest sequence identity to DmelGSTT1, although GF12485 and GF12486 show slightly greater identity to DmelGSTT2. There is a large cluster of Epsilon class GSTs located on scaffold 13266 as well as the Theta and Sigma class GSTs. FlyBase shows two D. ananassae GSTs; Dana\GF12160 and Dana\GF12161, as orthologs of GSTE1 of D. melanogaster with 78% and 76% sequence identity. GSTE2 also appears to have 2 D. ananassae orthologs, Dana\GF12162 (77% identity) and Dana\GF12168 (67% identity). Surprisingly, DmelGSTE3 was found to have 4 D. ananassae orthologs with 67% to 78% amino acid identity (Dana\GF12163, Dana\GF12165, Dana\GF12166 and Dana\GF12167; Table S1 in file S1). Dana\GF12164 is annotated to be an unknown GST but comparison of the translated protein to Epsilon class GSTs also shows it to be a GSTE3 ortholog with 67% identity (Table S1 and Figure S34E in file S1). So interestingly, D. ananassae has 5 GSTE3 orthologs. Four genes Dana\GF12171, Dana\GF12172, Dana\GF12173 and Dana\GF12174 show orthology to GSTE5, GSTE6, GSTE7 and GSTE8 of D. melanogaster with 61% to 75% amino acid identity (Table S1 in file S1). These 4 D. ananassae genes span about 3.4 kb, a similar distance to the 4 kb in D. melanogaster for the 4 Epsilon genes; however Dana\GF12171 and Dana\GF12172 appear to be more related to GSTE6 whereas Dana\GF12173 and Dana\GF12174 are more related to GSTE7. The 4 D. ananassae GSTs appear to share greater identity with the D. melanogaster GSTs than they do with each other, which suggests that gene divergence occurred after the tandem duplication events that generated the four genes.

#### Drosophila pseudoobscura

In D. pseudoobscura, there are large chromosomal rearrangements, compared to D. melanogaster, one of which moves the GST genes from chromosome 3L to the right arm of chromosome X. Therefore the GST Omega class members are found on chromosome XR_group6 instead of chromosome 3L. GSTT3 and GSTT4 are on different scaffolds, XL_group1e and XL_group3a, respectively. Searches with translated sequences from both Dpse\GA15569 and Dpse\GA24982 hit GSTT1 and GSTT2. Although, both Dpse\GA15569 and Dpse\GA24982 have greater identity against DmelGSTT1, 77% and 73%, respectively. Dpse\GA15569 shows the greater identity; therefore we have annotated Dpse\GA15569 as a GSTT1. Searches with Dpse\GA18009 protein sequence retrieved GSTD2, GSTD4 and GSTD5 of D. melanogaster with both GSTD2 and GSTD5 having 72% identity (Table S1 in file S1). Although Dpse\GA27027 and Dpse\GA27028 were not annotated to be orthologs to any D. melanogaster GST, a BLAST search of both sequences hit DmelGSTD2, GSTD4 and GSTD6. Although the GSTE3 ortholog of D. pseudoobscura, Dpse\GA14541 (73% amino acid identity), has been annotated, Dpse\GA24907 (67% amino acid identity) also appears to be an ortholog of GSTE3 (Table S1 in file S1). Dpse\GA14545 appears to be the ortholog of GSTE5 (76% identity), GSTE6 (76% identity), GSTE7 (68% identity) and GSTE8 (68% identity) with other D. pseudoobscura ortholog candidates showing <60% amino acid identity (Figure S34F in file S1). So these 4 DmelGSTs show the greatest orthology to this one DpseGST.

#### Drosophila persimilis

*D. persimilis* shares an analogous karyotype to *D. pseudoobscura* and both are classified in the obscura group [28]. *D. persimilis* also has been sequenced to low coverage which results in partial sequences for the *DperGSTZ2* and *GSTT4* genes. The gene organization of GST Delta class in *D. persimilis* is similar to that of *D. pseudoobscura.* A BLAST search with Dper\GL27300 hit GSTD2, GSTD4 and GSTD5 with 73%, 71% and 73% amino acid identities. The two proteins from genes *Dper\GL27301* and *Dper\GL27302* show the highest amino acid identity to DmelGSTD2 of 62% and 64%, respectively; although Dper\GL27302 also shows 64% identity to DmelGSTD4 (Table S1 in file S1). While FlyBase has annotated Dper\GL17770 to be GSTE3 with 72% amino acid identity, Dper\GL17772 also has its highest identity of 67% against GSTE3. A search with Dper\GL17773 protein hit GSTE5 (76% identity), GSTE6 (77%), GSTE7 (68%) and GSTE8 (68%). Dper\GL17152 has orthology to both GSTT1 (77% identity) and GSTT2 (69% identity) of *D. melanogaster*. *Dper\GL17151* would appear to be the remainder of the paired Theta GST genes that usually generate T1 and T2 (Figure S34G in file S1). However, in this species *Dper\GL17151* is a pseudogene that encodes a protein of 111 amino acids which is only the N-terminus half of the protein as discussed above in the Pseudogenes section.

#### Drosophila willistoni

There is a cluster of Delta class genes, *Dwil\GK11202*, *Dwil\GK11203* and *Dwil\GK11871-11876* that have been annotated as GSTs. Although, *Dwil\GK 11203* is a pseudogene as it encodes a protein of only 108 amino acids. These 7 genes share very high conservation of amino acid encoding sequence (58-65% identity) to one another as well as high identity (>58%) to GSTD1, GSTD2, GSTD4, GSTD5 and GSTD8 of *D. melanogaster* (Table S1 in file S1). The genome sequence of *D. willistoni* has several fragmentations so GSTT1 and GSTT2, GSTS1 and GSTE14, GSTE12 and the large cluster of Epsilon class GSTs are located on four different scaffolds (Figure S34H in file S1). FlyBase has annotated Dwil\GK22989 as the DwilGSTE7 ortholog with 64% identity to DmelGSTE7. However, both translated proteins from *Dwil\GK22990* and *Dwil\GK22991* show 61% to 69% identity to *D. melanogaster* GSTE5, GSTE6, GSTE7 and GSTE8. FlyBase does not show orthologs for Dwil\GK22988 to any isoform but BLAST searching hits GSTE3 with 60% amino acid identity. BLAST searching with both Dwil\22985 and Dwil\22987 hit DmelGSTE2 with 61% and 63% identity, respectively. *D. willistoni* is the only species that appears to lack the GSTE13 ortholog. Searches with Dwil\GK15953 and Dwil\GK15954 hit both GSTT1 and GSTT2, with both of their sequences showing greater identity to GSTT1 (Figure S34H and Table S1 in file S1).

#### Drosophila virilis

Although annotated as an unknown Delta class GST Dvir\GJ22852 shows>60% amino acid identity to GSTD1 (61%), GSTD2 (62%) and GSTD5 (63%). Dvir\GJ22854 also is annotated as an unknown Delta GST and shows ≥60% identity to GSTD1 (64%), GSTD2 (63%), GSTD4 (62%), GSTD5 (64%), GSTD8 (64%) and GSTD10 (60%). FlyBase does show orthology for Dvir\GJ22855 and Dvir\GJ22856 with GSTD2, GSTD4 and GSTD5. However, amino acid comparisons with D. melanogaster Delta class isoforms show a more extensive orthology. Dvir\GJ22855 shows ≥60% identity to GSTD1 (65%), GSTD2 (67%), GSTD4 (66%), GSTD5 (65%), GSTD8 (66%) and GSTD10 (61%). Dvir\GJ22856 shows ≥60% identity to GSTD1 (62%), GSTD2 (64%), GSTD4 (62%), GSTD5 (63%) and GSTD8 (60%). Such similar identities to multiple D. melanogaster isoforms makes it difficult to identify single orthologs, although it does give clues to GST evolution. FlyBase has not identified orthologs for GSTE1 and GSTE2. However, using DmelGSTE1 and DmelGSTE2 as template to BLAST search D. virilis, we identified 2 unknown GSTs on two different scaffolds. Dvir\GJ21357 and Dvir\GJ12879 show the greatest identity to GSTE1 and GSTE2, (58% and 68% identity) respectively (see Table S1 in file S1). FlyBase shows Dvir\GJ19892 with orthology to GSTE5 (61% identity), GSTE6 (60% identity), GSTE7 (61% identity) and GSTE8 (58% identity). Dvir\GJ19893 is also shown by FlyBase to be orthologous to GSTE5 (66% identity), GSTE6 (65% identity), GSTE7 (63% identity) and GSTE8 (61% identity). FlyBase shows Dvir\GJ21509 with orthology to both GSTT1 (68% identity) and GSTT2 (60% identity). In this species, there appears to be only 3 Theta genes and no GSTO1 ortholog (Figure S34I in file S1).

#### Drosophila mojavensis

Zeta and Delta class GSTs are located on scaffold 6540. The 2 Zeta genes are 1.4 kb apart and the Zeta 2 gene is alternatively spliced to generate 3 protein products as mentioned above. The Delta cluster appears to consist of only 7 genes with 2 genes being about 15 Mb distant from the others (Figure S34J in file S1). FlyBase shows *Dmoj\GI23195* with orthology to *DmelGSTD2*, *DmelGSTD4* and *DmelGSTD5* and a translated amino acid alignment shows identity as 63%, 58% and 64%, respectively. FlyBase also identified 2 unknown GSTs, *Dmoj\GI23193* and *Dmoj\GI23194*. Translated amino acid alignment shows high identity to multiple *D. melanogaster* Delta GSTs with>60% to GSTD1, GSTD2, GSTD4, GSTD5 and GSTD8 for both genes (Table S1 in file S1). FlyBase also identified *Dmoj\GI24379* and *Dmoj\GI22354* as orthologous to *DmelGSTD1* and a translated amino acid alignment shows 93% and 88% identity to DmelGSTD1, respectively. The proteins from these 2 genes share 92% identity to each other. Several different scaffolds contain the Epsilon, Sigma and Theta class GSTs (Figure S34J in file S1). *Dmoj\GI24095* is isolated on scaffold 6500 and FlyBase has identified it as a GST gene. We have translated and aligned the protein product to show it has the best match to DmelGSTE1 with 55% amino acid identity. FlyBase has both *Dmoj\GI16623* and *Dmoj\GI16624* as orthologs to *DmelGSTE2* and a translated amino acid alignment shows 64% and 66% identity, respectively. Dmoj\GI20122 and Dmoj\GI20123 have been annotated as being orthologs to GSTE5, GSTE6, GSTE7, and GSTE8 of *D. melanogaster*. However, both translated protein products have the greatest identity with DmelGSTE6 of 63% and 64%, respectively (Table S1 in file S1). *Dmoj\GI18422* is annotated to have orthology to both *GSTT1* and *GSTT2*, although a translated amino acid alignment shows greater identity to GSTT1, 64% versus 56%. Similar to *D. virilis*, there appears to be only 3 Theta GST genes and no GSTO1 ortholog to *D. melanogaster* in this species (Figure S34J in file S1).

#### Drosophila grimshawi

D. grimshawi is of special interest since there are 2 scaffolds, Sc_15074 and Sc_14830, which appear to contain the same 4 Delta GSTs genes. Moreover, Sc_15074 also contains a fifth GST, annotated as GSTD11, not on the second scaffold (Figure S34K in file S1). FlyBase has annotated two of the genes on each scaffold to be GSTD1 and GSTD10; however, the proteins of the 2 GSTD1 (Dgri\GH20186 and Dgri\GH13103) and 2 GSTD10 (Dgri\GH20197 and Dgri\GH13113), one from each scaffold, are 99% identical with a single amino acid difference between them. For GSTD1 the difference is Ser163Thr and for GSTD10 the difference is Ala76Thr. Both DgriGSTD1 proteins share 93% identity with DmelGSTD1 and both DgriGSTD10 proteins share 75% identity with DmelGSTD10. A third Delta gene pair (Dgri\GH20559 and Dgri\GH17521) has been annotated to be orthologous to GSTD2, GSTD4 and GSTD5 and this gene's protein from each scaffold is also 99% identical to the other scaffold product with the difference Val24Ile. Both protein products would share between 61% and 66% identity with GSTD1, GSTD2, GSTD4, GSTD5, GSTD8 and GSTD10 from D. melanogaster. The fourth Delta gene common to both scaffolds (Dgri\GH20548 and Dgri\GH17520) is annotated as an unknown GST. From both scaffolds this common gene would translate to a 100% identical protein. The protein product also has 60% to 64% amino acid identity with GSTD1, GSTD2, GSTD4, GSTD5, GSTD8 and GSTD10 from D. melanogaster. Uniquely among the 12 Drosophila species of this study D. grimshawi has been annotated to have 3 Zeta GST genes. Dgri\GH19140 is annotated as the GSTZ1 ortholog. Dgri\GH19138 is annotated to be GSTZ2 by FlyBase and as discussed above we have identified 3 alternatively spliced products similar to the other drosophilids. Interestingly, Dgri\GH19139 is located between GSTZ1 and GSTZ2 (Figure S34K in file S1). Moreover it possesses GST features and conserved sequence to Zeta class. The translated protein from Dgri\GH19139 shows 54% to 60% amino acid identity to the 4 Zeta proteins (GSTZ1, GSTZ2A, GSTZ2B and GSTZC) from D. melanogaster. This is actually a quite low identity comparison for Zeta proteins as these 4 proteins have 99% to 100% identity for the respective orthologs from the 12 drosophilids in this report. This suggests this extra Zeta GST has quite rapidly diverged as the grimshawi clade would have diverged from the virilis-repleta radiation only about 42.9 Ma [28], [32]. The virilis-repleta radiation contains both the D. virilis and D. mojavensis species. Epsilon class GST genes are located on several different scaffolds (Figure S34K in file S1). Scaffold 15245 contains a large cluster of Epsilon members as well as the Sigma class GST. Dgri\GH21669 is annotated to be orthologous to GSTE5 (63% identity), GSTE6 (66% identity), GSTE7 (56% identity) and GSTE8 (59% identity) from D. melanogaster. On scaffold 15126 where the annotated GSTE1 (Dgri\GH13569; 58% amino acid identity to DmelGSTE1) is located, there is also an annotated unknown GST (Dgri\GH13568) located ∼2 kb upstream of DgriGSTE1. Protein products from these 2 genes share 88% identity with each other, which also suggests rapid divergence after duplication. Amino acid translation of Dgri\GH22936 shows 70% sequence identity to DmelGSTT1 compared to 62% for DmelGSTT2. Again, there appears to be only 3 Theta GST genes and no GSTO1 ortholog to D. melanogaster in this species. Furthermore the GSTO2 gene encodes only one protein which has greater sequence identity to the DmelGSTO2B (74% identity) than to the GSTO2A variant (62% identity).

## Discussion

**Figure 2:**
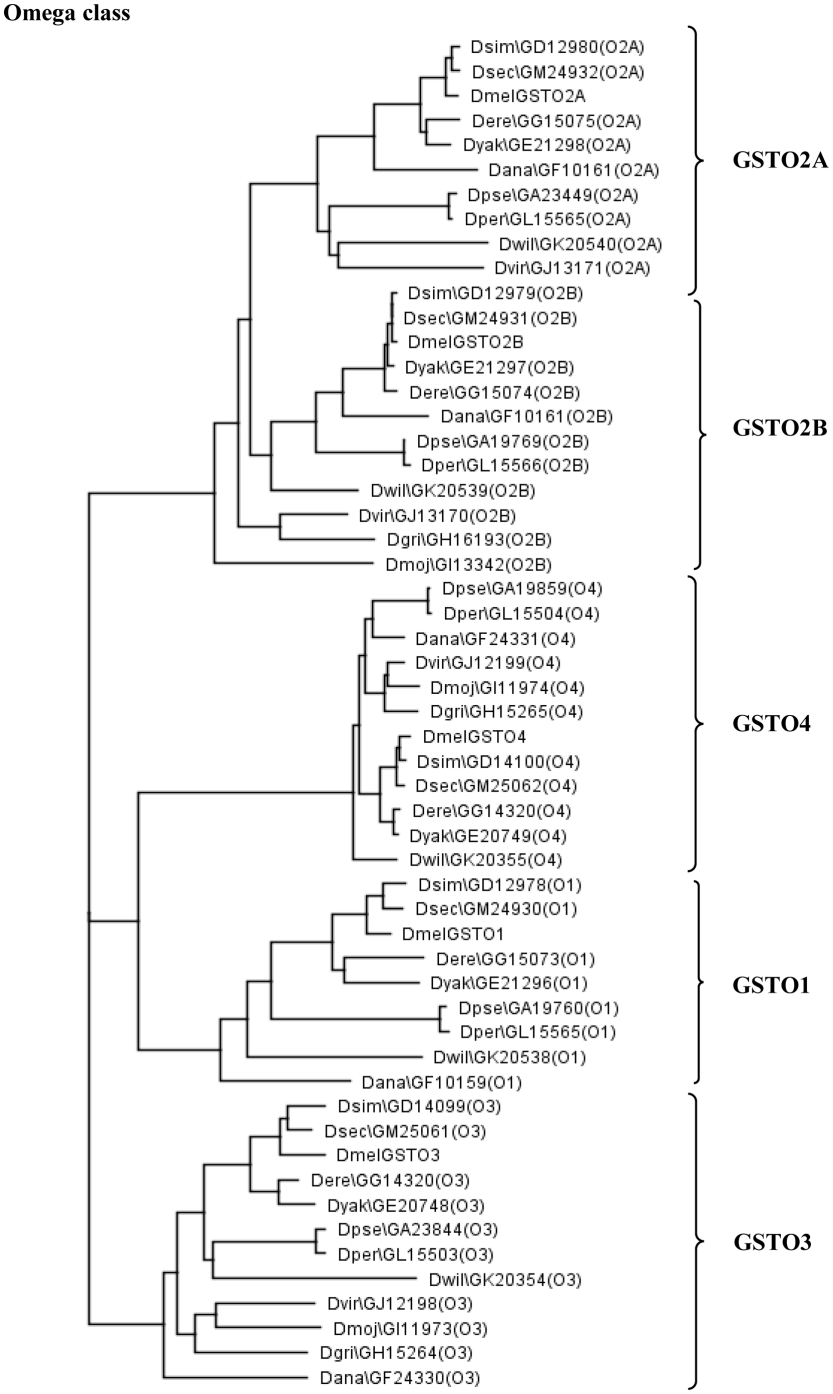
Phylogenetic tree analysis. The phylogenetic tree analysis used sequences from the Omega class from the 12 *Drosophila* species. Each isoform is designated by their FlyBase symbol followed by the *D. melanogaster* ortholog name in parenthesis. For example, Dana\GF10159(O1), where O1 refers to the DmelGST Omega 1 ortholog.

This comparative genomic study of the 12 *Drosophila* genomes has allowed classification of novel GSTs, several highly conserved GSTs as well as proteins unique to a lineage or single species. The analysis has also allowed a prediction of the evolutionary origin of the isoforms within a GST class. A parsimony tree analysis shows clustering of the designated isoforms for each GST class, with the best example being Omega class (Figure 2, for other GST classes see Figure S35 in file S1). In general the clustering of each GST isoform on the parsimony tree is similar to the fly species phylogenetic tree (Figure 1), which suggests a link between the GST isoforms and the fly species. This implies that as the fly species diverge to adapt to the new ecological niches the GST family played roles in this adaptation.

There are 19 orthologous GST genes that all 12 *Drosophila* species possess and these 19 genes express 23 proteins. Delta class consists of 11 genes (expressing 12 proteins) in *D. melanogaster* but orthologs for only 2 of these Delta genes (expressing 3 proteins) are present in all 12 *Drosophila* species. In contrast, of the 14 Epsilon class genes in *D. melanogaster* 8 orthologs are conserved across the 12 species. Enzymes from both classes have been proposed to play significant roles in detoxification of endogenous and xenobiotic compounds.

The Omega class GSTO1 is present in only the subgenus *Sophophora* species, possibly originating from a duplication of GSTO4 as these proteins share the greatest identity. The alternatively spliced GSTO2 gene in subgenus *Sophophora* generates 2 protein products but in 2 of the 3 *Drosophila* subgenus species generates only one protein. The GSTO2 gene appears to become alternatively splice during the virilis-repleta divergence as it is spliced in *D. virilis* but not *D. mojavensis*. Curiously, the intron-exon spliced gene organization is the same for *D. virilis* (subgenus *Drosophila*) as it is for the 9 subgenera *Sophophora* species; which suggests that the spliced product was lost in the other two *Drosophila* subgenus species.

The 12 species of this study possess 3 of the 4 alternatively spliced genes. Even though the alternatively spliced GSTO2 gene is not present in *D. mojavensis* and *D. grimshawi* the coding sequence for the GSTO2B protein is still present. The remaining spliced products tend to be highly conserved among the 12 species. The best example is the 3 protein splice products from the GSTZ2 gene with the 11 species orthologs possessing 94% to 100% amino acid identity to the relevant *D. melanogaster* proteins. However the alternatively spliced proteins also show us that the spliced products can evolve or diverge at different rates, as demonstrated by the subgenera *Drosophila* species GSTT3A and B isoforms. We also see for single gene products that although the gene may be present and conserved across the 12 species it can still diverge significantly as shown by GSTZ1 with 54% to 95% amino acid identity compared to *D. melanogaster*. In contrast to this divergence some of the orthologous genes present in all 12 species can be highly conserved as shown by GSTD1 with 93% to 98%, and GSTS1 with 92% to 99% amino acid identity compared to *D. melanogaster*. Obviously, the proteins that are so highly conserved must perform a critical or important function to be maintained over more than 60 million years of species divergence and evolution. Unfortunately the specific functions of the GSTs are unknown at this time. Although it is interesting to note that DmelGSTD1 was found to have a very broad substrate specificity [14].

Both GSTT4 and GSTT3A occur in all 12 *Drosophila* species and the 2 proteins show low conservation between them. This suggests an ancient duplication and divergence for the 2 genes. We suggest that DmelGSTT3A appears to be most related to the ancestral Theta GST as it shows the greatest amino acid identity to the single Theta GSTs in the Hymenoptera *Apis mellifera* (honey bee), 41%, and Lepidoptera *Bombyx mori* (silkworm moth), 44%. DmelGSTT3A also has 46% identity with one of the 2 Theta GSTs in the Coleoptera *Tribolium castaneum* (red flour beetle). DmelGSTT3A also has the greatest identity, 51%, to the mosquito *Anopheles gambiae* GSTT1 and 41% to the other *A. gambiae* Theta, GSTT2. The Diptera fruit flies and mosquitoes are estimated to have diverged 238.5 to 295.4 Ma [33]. The GSTT3 gene would have become alternatively spliced before the *Drosophila* divergence as both GST3A and GST3B appear in all 12 species with reasonably high conservation. GSTT2 appears to have duplicated after subgenera *Drosophila* and *Sophophora* diverged at 62.9Ma but before the willistoni, obscura and melanogaster groups diverged at 62.2 Ma. GSTT2 most likely originated from a GSTT1 duplication event as GSTT2 still shows the highest identity towards GSTT1.

Although some of the gene copies clearly originate with tandem duplication events such as for the copies of GSTD10 and GSTE1 in *D. erecta*, GSTE3 in *D. yakuba*, or GSTD7 and GSTE3 in *D. ananassae*. Analysis also indicates that multiple duplication events occurred from a single gene template. A clear example is the 5 copies of the orthologous GSTE3 in *D. ananassae* with 59% to 78% amino acid identity between the 5 proteins, demonstrating that these proteins have also undergone diversification after duplication. Several of the GSTs are lineage specific which suggests that the genes appeared after species divergence. GSTE3, GSTO1 and GSTT2, appear only in the *Sophophora* genus which would place their earliest appearance at about 62.9 Ma. The Delta class GSTD7 appears only in the Melanogaster group which diverged about 54.9 Ma. Both the Epsilon GSTE8 and Delta GSTD3 appear only in the Melanogaster subgroup which diverged about 44.2 Ma. The GSTD3 appears to have originated from a Delta 2/4/5 gene and rapidly diverged during the speciation of the Melanogaster subgroup. Analysis of the 12 species data suggests this Delta 2/4/5 gene to have originated with GSTD1 and then the 2/4/5 gene further duplicated to yield the other Delta genes, excluding GSTD11 which seems to be concurrent with GSTD1 in the *Drosophila* genus. In fact, consideration of the data from the 12 species suggests that GSTD1 gave rise to a gene template that further duplicated to initially yield the Delta 2/4/5/8/10 genes. This can be observed as the template gene is still present in several of the species, sometimes as multiple copies. Although there are many conserved Epsilon GSTs in these 12 *Drosophila* species the template gene scenario also can be observed with the template gene Epsilon 5/6/7/8 being found in several species again sometimes as multiple copies. The multiple copies would obviously be duplications and possibly in the process of diverging as shown by the 4 copies of Epsilon 5/6/7/8 in *D. ananassae* (Table S1 in file S1).

**Figure 3:**
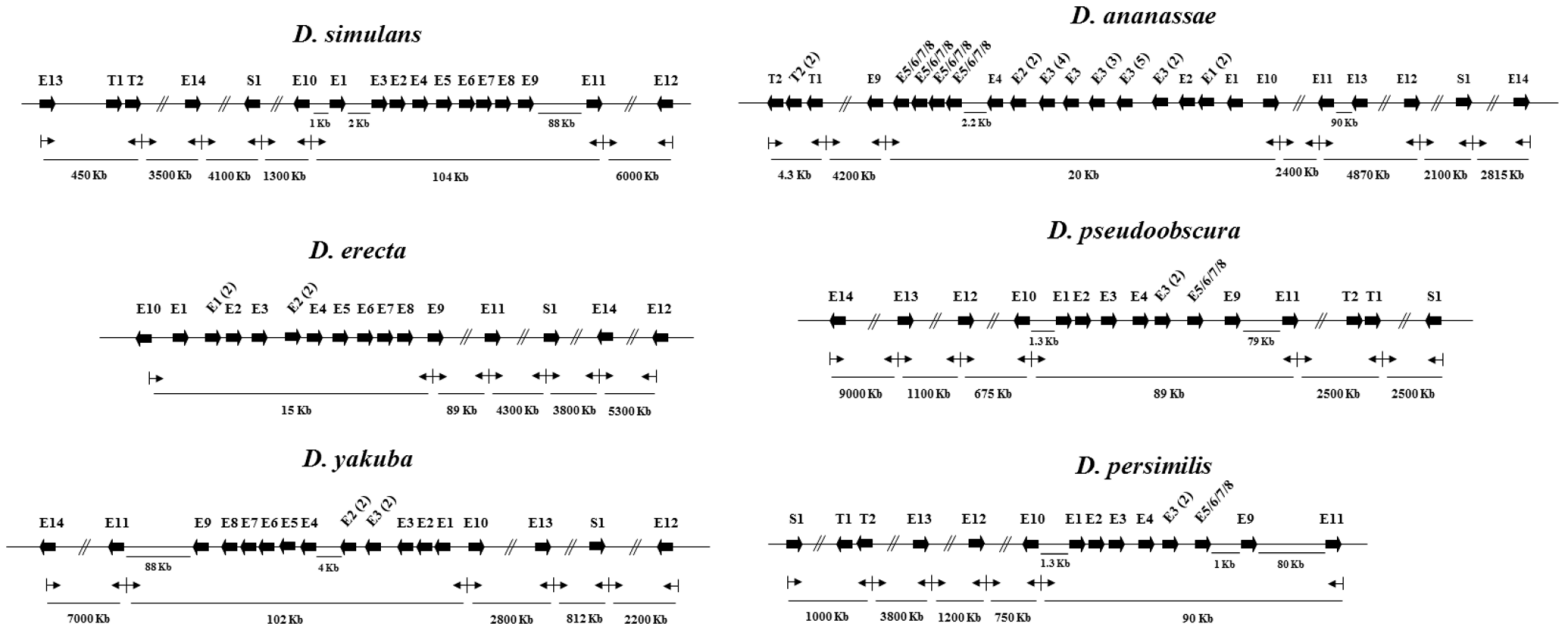
Epsilon GST gene cluster and adjacent region from six *Drosophila* species. Genomic maps showing the Epsilon GST gene organization in six *Drosophila* species. These examples illustrate the overall synteny of the Epsilon gene cluster as well as the micro-rearrangements that occur in the individual species. The genes are shown as arrows which represent the direction of transcription.

In addition to the well-known major chromosomal rearrangements, both paracentric and pericentric inversions as well as chromosomal fusions [28] (and references therein), the regions containing the GST genes also appear to have undergone micro-rearrangements. The sizes of these rearranged pieces are much smaller than the usually analyzed synteny blocks that are measured in megabases. These small pieces can contain intronless protein coding sequences and may be only a few hundred base pairs in length. The GST genome maps for the 11 *Drosophila* species illustrate the micro-plasticity of the GST gene region (Figure S34 in file S1). For example, the extreme rearrangement of the Epsilon cluster as observed for *D. grimshawi* where it appears the Epsilon genes have been dispersed compared to the other species (Figure S34K in file S1). Many of these rearrangements in and around the Epsilon gene cluster in six species are shown for illustrative purposes (Figure 3). One obvious rearrangement is that the Epsilon cluster, approximately 20 Kb, has undergone an inversion such as in *D. yakuba, D. ananassae* and *D. virilis*, relative to the surrounding GST genes (Figure 3; Figure S34D, E, I in file S1). We also observed multiple duplications from the same gene template, clearly shown in *D. ananassae*, as well as rearrangements within the cluster as shown by *D. simulans* (Figure 3 and Table S1 in file S1). Another reorganization noted is that the GST genes around the Epsilon cluster appear to have been ‘shuffled’ as for *D. erecta, D. pseudoobscura, D. persimilis* and *D. mojavensis* (Figure 3; Figure S34C, F, G and J in file S1). This shuffling is evident even between closely related species pairs such as *D. erecta* and *D. yakuba* which diverged about 10.4 Ma or the *D. pseudoobscura* and *D. persimilis* species pair more recently diverged at about 0.85 Ma. There also appears to be inversion of individual GST genes or groups of GST genes relative to the Epsilon cluster but still remaining near the cluster. For an illustration of these points consider Sigma *GSTS1* from the different species, its orientation and position relative to the Epsilon cluster gene *GSTE11* (Figure 3). For example, in *D. erecta* transcription of *GSTS1* and *GSTE11* is in the same direction with no GST genes in-between and downstream of *GSTS1* are *GSTE14* (3800 Kb away) and *GSTE12* (9100 Kb away). However, in the sister species *D. yakuba*, *GSTS1* and *GSTE11* are transcribed in opposite directions as the Epsilon gene cluster is ‘inverted’. Also in *D. yakuba GSTE13* is between the Epsilon gene cluster and *GSTS1* whereas *GSTE12* is only 2200 Kb downstream of *GSTS1* with *GSTE14* on the opposite side of the Epsilon gene cluster.

We report here the identification of new GSTs as well as improved annotation of the GSTs in the FlyBase genomes database of 12 *Drosophila* species. This analysis has also highlighted several salient features of the compared genomes. The GST gene family is ancient and in the ‘recently’ evolved *Drosophila* genus has undergone repeated local duplications followed by transposition, inversion, micro-rearrangements and functional diversifications of these copies (Figure S34 in file S1). The gene copies originate from tandem duplications as well as multiple duplications from a single gene template. In the 12 species of this study, we identified several GST proteins that are highly conserved across the 63 Ma of speciation; as well as several GSTs that are lineage specific or apparently species specific (Figure S34 and Table S1 in file S1). Although many of the GST genes show clear orthology across the *Drosophila* species the genomic organization of the genes is also clearly very flexible across the species. The co-linearity, relative positions and orientations of the GST genes, within a relatively short genomic distance, appear to be unique in each species; which shows that these genomic rearrangements have occurred multiple times over the course of evolution and divergence of the 12 species in the last 60 million years. Although the mechanism of the genome micro-plasticity is currently unknown, it appears to have contributed substantially to the evolution of a gene family. Whether this micro-plasticity is a unique feature of *Drosophila* species or a general property of eukaryote genomes remains to be shown.

## Materials and Methods

### Comparative genomic analysis

Our analysis is the genomic comparison of 11 *Drosophila* species to *D. melanogaster*. The *D. melanogaster* GST genes and their translated proteins were used as a benchmark template to annotate the GST orthologs in the other sequenced *Drosophila* genomes. We retrieved the sequences from FlyBase version FB2013_05, released September 13, 2013. This version is based on the Annotation Release 5.53 of *D. melanogaster*, Annotation Release 3.1 of *D. pseudoobscura*, Annotation Release 1.4 of *D. simulans*, Annotation Release 1.3 of *D. ananassae, D. erecta, D. grimshawi, D. mojavensis, D. persimilis, D. sechellia, D. willistoni* and *D. yakuba* and Annotation Release 1.2 of *D. virilis*. Each single GST gene has been analyzed by manual curation. Briefly, the GST genes of *D. melanogaster* were first used for BLASTn searching. Then GSTs that cannot be obtained from the first screen were searched for again by using amino acid sequence of *D. melanogaster* as a query sequence for the respective species. Moreover we also searched for putative GST genes by their molecular function. The retrieved sequences were then confirmed by nucleotide and amino acid sequence alignment using the ClustalX and GeneDoc programs.

### Alternative splicing determination

The genome region of each *Drosophila* species was retrieved from Flybase. To determine whether the GSTs from the other 11 *Drosophila* species also undergo alternative splicing as in *D. melanogaster*, the splicing patterns of DmelGSTZ2_A/B/C_, D11_A/B_, T3_A/B_ and O2_A/B_ for introns and exons as well as the protein amino acid sequences were used as templates and compared to the other 11 *Drosophila* species for each gene.

### Phylogenetic tree construction

The GST sequences of all 12 *Drosophila* species from the same class were used for analysis. The trees were constructed by Geneious R7 program. BLOSUM62 was used to score the alignments. Distances were obtained from pairwise alignments of all sequence pairs and calculated using Jukes-Cantor algorithm. The phylogenetic trees were constructed by neighbor-joining method with a gap penalty of 1.

### GST annotation and nomenclature

The FlyBase symbols are unique identifiers and are therefore used throughout this report for clarity. Additionally, we employ the GST nomenclature for the 11 *Drosophila* species which is based on the *D. melanogaster* GST system. We have assigned a descriptive name to all GST orthologs as well as the 2 letter ID followed by the 5 digit number. The uppercase D refers to *Drosophila* followed by the first 3 letters of each species' name, GST class designation and individual number. For example, Dsim\GD17126 is *D. simulans*, this gene was found to be orthologous to *DmelGSTT4* the *D. melanogaster* GST numbered 4 from Theta class. Therefore the name of this gene would be *DsimGSTT4* and the expressed protein product would be DsimGSTT4.

## Supporting Information

File S1 **Supplementary Figures and Tables.** Figure S1. The annotated gene region of *Dsim\GD17126* (*DsimGSTT4*) from Flybase. A. 6 exons of *DsimGSTT4* code for a protein of 288 amino acids. If the putative exons 2 and 3 are intron as in *DmelGSTT4*, *DsimGSTT4* would encode a GST protein of 237 amino acids. B. The GSTT4 amino acid alignment of the proposed *D. simulans* and *D. melanogaster* shows 100% identity. Figure S2. The annotated gene region of *Dere\GG21888* (*DereGSTE6*) from Flybase. A. The underlined nucleotides at the 5′end is the excluded sequence which makes *DereGSTE6* larger than usual for Epsilon class GSTs. Exclusion of these 54 nucleotides and starting the translation from the second ATG start site as shown in red, *DereGSTE6* will code for a GST protein of 222 amino acids. B. The GSTE6 amino acid sequence alignment of *D. erecta* and *D. melanogaster* shows 93% identity. Figure S3. The annotated gene region of *Dana\GF17942* from Flybase. A. A possible ATG start site is situated in the intron region as shown by the underline and then read through the second exon. The coding sequence of *Dana\GF17942* will become one single exon and code for a protein of 220 amino acids. B. The amino acid sequence alignment of Dana\GF17942 and DmelGSTD2 shows 64% identity. Figure S4. The annotated gene region of *Dana\GF11968* (*DanaGSTE11*) from Flybase. A. 45 nucleotides of the coding sequences of *DanaGSTE11* including stop codon are in the intron region. Including these 45 nucleotides as the last part of exon 2, *DanaGSTE11* will be a 225 GST protein. B. The GSTE11 amino acid sequence alignment of *D. ananassae* and *D. melanogaster* shows 87% identity. Figure S5. The annotated gene region of *Dvir\GJ22855* from Flybase. A. The suggested ATG start site is 48 nucleotides (amino acids encoded are shown below) upstream of exon 1. This now larger exon is in-frame and contiguous and encodes a protein of 216 amino acids. B. The amino acid sequence alignment of our curated Dvir\GJ22855 and DmelGSTD2 shows 67% amino acid identity. The arrow indicates the previously annotated transcriptional start site methionine (M). Figure S6. The annotated gene region of *Dvir\GJ22856* from Flybase. A. The possible ATG start site is 33 nucleotides (amino acids encoded are shown below) upstream of exon 1. This larger exon is in-frame and contiguous and encodes a protein of 211 amino acids. B. The amino acids sequence alignment of Dvir\GJ22856 and DmelGSTD2 shows 64% amino acid identity. The arrow indicates the previous annotated methionine (M). Figure S7. The annotated gene region of *Dvir\GJ23571* (*DvirGSTZ1*) from Flybase. A. The possible ATG start site is 255 nucleotides upstream of exon 1 as shown in underline. This larger exon is in-frame and contiguous and codes for a protein of 247 amino acids. B. The GSTZ1 amino acids sequence alignment of *D. virillis* and *D. melanogaster* shows 61% identity. The arrow indicates the previous annotated methionine (M). Figure S8. The annotated gene region of *Dsec\GM23038* (*DsecGSTT3*) from Flybase. A. The incomplete gene sequencing of *D. sechellia GSTT3* shown as Ns in the middle of the gene. B. The amino acid sequence alignment of GSTT3 *D. sechellia* and *D. melanogaster* is highly conserved. Figure S9. The annotated gene region of *Dper\GL26999* (*DperGSTT4*) from Flybase. A. We have proposed that exons 2 and 3 of annotated *DperGSTT4* are intron and the remainder of the coding sequence is in the incompletely sequenced region and exons 4, 5 and 6. The sequence of exons 5 and 6 is quite conserved compared to the last 2 exons from *D. melanogaster*. The new sequence of *DperGSTT4* and the incomplete part are shown as underline. This gene needs to be re-sequenced to obtain the missing sequence. B. The amino acid alignment of GSTT4 *D. persimilis* and *D. melanogaster* shows high identity to each other. Figure S10. The annotated gene region of *Dper\GL13668* (*DperGSTZ2*). A. ATG start codon (as underline) of *GSTZ2B*, *Z2C* and *Z2A* are on exon 1, 2 and 3 respectively. They share exons 4 and 5. Exons 2 and 3 are intron of *GSTZ2B*, and exon 3 is intron of *GSTZ2C*, therefore the incomplete sequencing data does not affect the sequence translation of these isoforms. Fortunately, the coding regions of *GSTZ2B* and *Z2C* were sequenced. As their sequences are quite conserved with *D. melanogaster* they can be manually curated. B. Based on *D. melanogaster*, this gene would have 6 exons. But we found a single guanosine (G) insertion (shown in red) which causes a frameshift mutation. If the extra G is absent and the 59 nucleotides after this G are intron (double underlined) as in *D. melanogaster*, the DperGSTZ2A, Z2B and Z2C will show 96, 100 and 100% amino acid sequence identity to all 3 spliced products of DmelGSTZ2, respectively. Obviously, with the Ns in intron 2 and this extra G in exon 5 the gene should be sequenced again. Figure S11. The annotated gene region of *Dsec\GM24019* from Flybase. A. The *Dsec\GM24019* gene is located next to *Dsec\GM24018* gene on the same chromosome. There is a 149 nucleotide gap between the 2 genes. Although 69 nucleotides upstream of the annotated ATG start site of *Dsec\GM24019* (underline) code for GST conserved sequence, this GST is still too short be an active enzyme. B. The short amino acid sequence of Dsec\GM24019 shows 52% identity to the C-terminus part of DmelGSTD6. Figure S12. The annotated gene region of *Dsec\GM21877* from Flybase. A. Gene region of *Dsec\GM21877*. The coding sequence is 447 bp. B. The nucleotide sequence alignment of *Dsec\GM21877* and *Dmel\CR43687* pseudogene. Figure S13. The annotated gene region of *Dyak\GE11955* (*DyakGSTE1*) from Flybase. A. *Dyak\GE11955* was reported to have 1 intron. However the sequence of the annotated intron codes for part of the conserved DmGSTE1 protein. B. The amino acid sequence alignment of DmGSTE1 and DyakGSTE1, (1) and (2). DyakGSTE1 (1) is the protein sequence reported by Flybase. DyakGSTE1 (2) includes the protein coding sequence in the annotated intron region. Figure S14. The annotated gene region of *Dana\GF17941* from Flybase. Dana\GF17941 shows high amino acid sequence identity to the C-terminus part of DmelGSTD2, 4 and 5. Figure S15. The annotated gene region of *Dper\17151* (*DperGSTT2*) from Flybase. A. Gene region of *DperGSTT2* which codes for protein 212 amino acids. The sequence show high conservative to *DmelGSTT2*. B. The amino acid sequence alignment of DmelGSTT2 and the pseudogene product DperGSTT2. Figure S16. The annotated gene region of *Dper\GL26929* from Flybase. A. The gene region of *Dper\GL26929* codes for a protein of 163 amino acids. B. The amino acid sequence alignment of Dper\GL26929 to DmelGSTT1 and T2 show 27% and 29% amino acid identity. The conserved sequences are shown underlined. Figure S17. The annotated gene region of *Dwil\GK11203* from Flybase. A. Exon 1 is the annotated coding sequence from Flybase but we found conserved blocks of sequences located upstream of the annotated ATG start site as shown in the bracket. B. Dwil\GK11203 (1) is the annotated sequence reported by Flybase. It shows the highest amino acid sequence identity to DmelGSTD5 but it has about 60 critical amino acids missing from the active site so would not be an active GST enzyme. Therefore this gene is suggested to be a pseudogene. Dwil\GK11203 (2) shows some conserved sequence upstream of the annotated ATG start site. Figure S18. The annotated gene region of *Dvir\GJ24387* (*DvirGSTD10*) from Flybase. A. *Dvir\GJ24387*has been reported to have 2 exons and 1 intron. Although we found that part of the intron sequence (underlined) also codes for conserved sequence of DmGSTD10, this protein is still too short to be an active GST enzyme. The conserved sequence is QYGKDSTLYPKDIQTQALIN. B. The short amino acid sequence of DvirGSTD10 shows 49% amino acid sequence identity to DmelGSTD10. Figure S19. The annotated gene region of *Dvir\GJ19066* from Flybase. A. *Dvir\GJ19066* encodes a protein of 54 amino acids which shows the greatest amino acid identity to DmelGSTD1. Moreover we found possible conserved sequence of 63 nucleotides upstream contiguous with the annotated ATG start site (underlined). B. The short amino acid sequence of Dvir\GJ19066 shows 20% identity to the N-terminus part of DmelGSTD1. Figure S20. The annotated gene region of *Dsim\GD17492* (*DsimGSTT3*) from Flybase. A. The underlined ATG (exon 1 and exon 4) represent the start codons of T3B and T3A, respectively. The sequence from exons 5 to 6 of the *DsimGSTT3* gene comprises the sequence of exon 5 in *D. melanogaster*. Due to the first cytosine (C) of exon 6 is missing; the annotation split the sequence into 2 exons apparently to keep the remaining sequence in frame. Thus *DsimGSTT3A* and *T3B* are shorter than T3A and T3B in other species. B. DsimT3 (1) refers to the sequence reported by Flybase. DsimT3 (2) refers to the sequence from the new curation. If the missing cytosine is replaced, T3A and T3B will translate to proteins of 228 and 268 amino acids and show 98 and 97% amino acid sequence identity to *D. melanogaster*. Figure S21. The annotated gene region of *Dsim\GD11388* (*DsimGSTE11*) from Flybase. A. The curated ATG start site is highlighted in yellow, however the end of intron 1 shows a stop codon (TAG) in the annotation but which may be CTG, as in *D. melanogaster*, in this low coverage genome. B. The figure shows the amino acid alignment of DmelGSTE11, DsimGSTE11 (1) and DsimGSTE11 (2). DsimGSTE11 (1) is the annotated sequence from Flybase and DsimGSTE11 (2) is a new curated sequence. If the stop codon (TAG) at the end of intron 1 is CTG as in *D. melanogaster* and the translation start site is the yellow highlighted sequence, the proteins show 97% sequence identity to each other. The Ser13 in DmelGSTE11 (shown by arrow) is the catalytic serine and that region is generally conserved for active site topology which suggests that DsimGSTE11 (1) may be an inactive enzyme. Figure S22. The annotated gene region of *Dsim\GD24922* (*DsimGSTE12*) from Flybase. A. Using *DmelGSTE12* as template, we found that possibly a thymidine (T) is missing as shown by the underlined gap in exon 2. If this is so, intron 2 also would be translated to the coding sequence. If the thymidine is present a transcription read through would include intron 2 as coding sequence, which then would encode a protein of 223 amino acids. B. The panel shows the amino acid alignment of DmelGSTE12, DsimGSTE12 (1) and DsimGSTE12 (2). DsimGSTE12 (1) is the annotated sequence from Flybase and DsimGSTE12 (2) is a new curated sequence. The new curated sequence of DsimGSTE12 (2) aligns with DmelGSTE12 giving 98% amino acid sequence identity. Figure S23. The ambiguous annotation of *Dsec\GM24014* and *Dsec\GM24015* (*DsecGSTD2*). A. The first exon is the annotated coding sequence of *Dsec\GM24014* whereas the second exon belongs to *Dsec\GM24015* and the small gap of 23 nucleotides between the 2 genes is shown underlined. A missing cytosine of *Dsec\GM24014* results in a frame shift which also results in a premature stop codon (TGA). The addition of cytosine to *Dsec\GM24014* followed by a read through the gap as well as the exon of *Dsec\GM24015* would result in a protein of 215 amino acids. This single exon would encode GSTD2 of *D. sechellia*. B. The GSTD2 amino acids sequence alignment of *D. sechellia* and *D. melanogaster* shows 98% identity. Figure S24. The annotated gene region of *Dsec\GM25062* (*DsecGSTO4*) from Flybase. A. Using *DmelGSTO4* as template, we found that intron 2 and part of intron 3 (as underlined) of *D. sechellia GSTO4* show very highly conserved gene sequences to *DmelGSTO4*. However there are two insertions of cytosine, one in exon 2 the other in exon 3 as shown in red. The insertion in exon 2 would lead to a stop codon in intron 2 (TGA as underlined), hence its intron annotation. Moreover, annotated intron 2 contains 1 nucleotide less than *DmelGSTO4*. This frame shift leads to wrong coding protein. Therefore intron 2 and part of intron 3 were annotated by FlyBase as intron which makes *DsecGSTO4* shorter than normal GST Omega class (204 instead of 240 to 250 amino acids). The sequence of intron 3 that is underlined should be part of the protein coding gene, WCERLELLKLQRGEDYNYDESRFPQL. B. The comparison between the conserved block of *D. sechellia* and *D. melanogaster GSTO4*. In the annotated intron 2, 7 nucleotides downstream from the stop codon there appears to be a deletion (position shown in panel A as underlined T). If in the exon this one nucleotide deletion would result in a frame-shift. C. The figure shows amino acid alignment of GSTO4 from *D. sechellia* and *D. melanogaster*. The empty blocks are the missing sequences of intron 2 and 3. D. The figure shows amino acid alignment of GSTO4 from *D. sechellia* and *D. melanogaster* if the inserted cytosines are absent from exons 2 and 3, as in *D. melanogaster*, and one more nucleotide present in intron 2 (GTGATCTGGA**TC**CCTTCTGGAGCGGCCTGGA C**G**TCTACGAAAG), the full coding sequence would be present. The proteins would have 97% amino acid sequence identity to each other. This sequence is ambiguous because of the low coverage genome sequencing. Figure S25. The annotated gene region of *Dsec\GM21874* (*DsecGSTE1*) from Flybase. A. A possible ATG start site is located in the intron region. However it includes a TAG stop codon due to the absence of a T, TA(T)G. If the T is present as it is in *D. melanogaster*, the gene will code for a 206 amino acid protein. B. The GSTE1 amino acid sequence alignment of *D. sechellia* and *D. melanogaster* shows 65% identity. Figure S26. The annotated gene region of *Dsec\GM21098* (*DsecGSTE13*) from Flybase. A. In *D. melanogaster* the sequences from exon 3 to 4 are one continuous exon. There is one adenosine (A) insertion as shown in red which results in a frameshift. Therefore it appears some of the exon region was annotated as intron to keep the rest of the sequence in-frame. B. DsecE13 (1) refers to the sequence reported by Flyabse. DsecE13 (2) refers to the sequence from our new curation. The amino acid sequence alignment of DmelGSTE13 and DsecGSTE13 (1) shows 92% identity. The missing gap is the part of the gene which is annotated as intron. If the extra adenosine is absent as in D. melanogaster, the amino acid identity increases to 98% for 226 amino acids. Figure S27. The annotated gene region of *Dvir\GJ14962* (*DvirGSTE13*) from Flybase. A. We found one adenosine (A) insertion at nucleotide position 419 of exon 4 (shown in red). This insertion makes a frame shift which reads through the stop codon (TAA) shown in red. B. The figure shows the amino acid alignment of DmelGSTE13, DvirGSTE13 (1) and DvirGSTE13 (2). DvirGSTE13 (1) is the annotated sequence from Flybase and DvirGSTE13 (2) is a new curated sequence. The amino acid alignment of DmelGSTE13 and DvirGSTE13 (1) shows 57% identity. If the extra A is absent, as in D. melanogaster, the TAA stop codon shown in red will be in-frame. The two proteins then show 74% amino acid identity to each other. Figure S28. Alternative splicing scheme of *DyakGSTD11*. A. *DyakGSTD11* gene undergoes alternative splicing to generate 2 variants, *DyakGSTD11A* and *DyakGSTD11B*. *DyakGSTD11A*are from exon 1, 3, 4 and 5. The whole exon 1 and the first 10 nucleotides of exon 3 are proposed to be 5′UTR of *DyakGSTD11A* whereas the first 17 nucleotides of exon 2 is proposed to be 5′UTR of *DyakGSTD11B* as shown underlined. *DyakGSTD11A* and *DyakGSTD11B* share the sequences in exon 4 and 5. They also share 3′UTR which is the last 94 nucleotides of exon 5 (underline). B. We propose the possible 5′UTR of *DyakGSTD11A*. This sequence shows nucleotide sequence identity of 83% to *D. melanogaster*. C. We propose the possible 5′UTR of *DyakGSTD11B*. This sequence shows nucleotide sequence identity of 100% to *D. melanogaster*. D. We propose the possible 3′UTR of *DyakGSTD11*. This sequence shows nucleotide sequence identity of 81% to *D. melanogaster*. Figure S29. Alternative splicing scheme of *DyakGSTZ2*. A. *DyakGSTZ2* gene undergoes alternative splicing to generate 3 variants, *DyakGSTZ2A*, *DyakGSTZ2B* and *DyakGSTZ2C*. The first 356 nucleotides of exon 1 is proposed to be 5′UTR of *DyakGSTZ2B* as shown underlined. The first 40 nucleotides of exon 7 and the first 29 nucleotides of exon 6 are proposed to be 5′UTR of *DyakGSTZ2C* and *DyakGSTZ2A*, respectively. B. We propose the possible 5′UTR of *DyakGSTZ2A*. This sequence shows nucleotide sequence identity of 100% to *D. melanogaster*. C. We propose the possible 5′UTR of *DyakGSTZ2B*. This sequence shows nucleotide sequence identity of 89% to *D. melanogaster*. D. We propose the possible 5′UTR of *DyakGSTZ2C*. This sequence shows nucleotide sequence identity of 95% to *D. melanogaster*. E. We propose the possible 3′UTR of *DyakGSTZ2A*. This sequence shows nucleotide sequence identity of 83% to *D. melanogaster*. F. We propose the possible 3′UTR of *DyakGSTZ2B*. This sequence shows nucleotide sequence identity of 86% to *D. melanogaster*. G. We propose the possible 3′UTR of *DyakGSTZ2C*. This sequence shows nucleotide sequence identity of 85% to *D. melanogaster*. Figure S30. Alternative splicing scheme of *DyakGSTT3*. A. *DyakGSTT3* gene undergoes alternative splicing to generate 3 variants, *DyakGSTT3A*, *DyakGSTT3B* and *DyakGSTT3C*. The first 235 nucleotides of exon 1 is proposed to be 5′UTR of *DyakGSTT3B* as shown underlined. Exon 1, 2, 3 and the first 29 nucleotides of exon 4 is proposed to be 5′UTR of *DyakGSTT3C*. Exon 3 and the first 29 nucleotides of exon 4 is proposed to be 5′UTR of *DyakGSTT3A* as shown in yellow highlight. All 3 variants share exon 4–6 and the 3′UTR which is the last 165 nucleotides of exon 6 as underlined. B. We propose the possible 5′UTR of *DyakGSTT3B*. This sequence shows nucleotide sequence identity of 71% to *D. melanogaster*. C. We propose the possible 5′UTR of *DyakGSTT3C*. This sequence shows nucleotide sequence identity of 80% to *D. melanogaster*. D. We propose the possible 5′UTR of *DyakGSTT3A*. This sequence shows nucleotide sequence identity of 94% to *D. melanogaster*. E. We propose the possible 3′UTR of *DyakGSTT3*. This sequence shows nucleotide sequence identity of 72% to *D. melanogaster*. Figure S31. Alternative splicing scheme of *DyakGSTO2*. A. Flybase has reported that Dyak\GE21297 and Dyak\GE21298 are GST proteins in Omega class translated from 2 different genes. Our manual curation based on *D. melanogaster* has shown that these 2 GST proteins could possibly originate from the same gene which has undergone alternative splicing to yield 2 final protein products which are DyakGSTO2A and DyakGSTO2B. The two proteins share sequence in exon 1. The underlined sequence in exon 1 is proposed to be 5′UTR whereas the underlined sequences in exon 2 and 4 are proposed to be 3′UTR of *DyakGSTO2B* and *DyakGSTO2A*, respectively. B. We propose the possible 5′UTR of *DyakGSTO2*. Using D. melanogaster as a template this sequence shows 92% nucleotide sequence identity. C. We propose the possible 3′UTR of *DyakGSTO2A*. Using D. melanogaster as a template this sequence shows 70% nucleotide sequence identity. D. We propose the possible 3′UTR of *DyakGSTO2B*. Using D. melanogaster as a template this sequence shows 77% nucleotide sequence identity. Figure S32. The curation for alternative splicing in *D. yakuba*. Our curation of alternative splicing shows all 11 drosophila had alternatively spliced genes. This figure uses the *D. yakuba* genes to illustrate this result. The details for this cartoon are given in Table S2. Figure S33. The alternatively spliced GST proteins from the 12 *Drosophila* species. Each identified alternatively spliced GST from *D. melanogaster* was used to manually curate the other 11 *Drosophila* species. This figure shows the amino acid alignment for the spliced GSTs with a matrix table beneath showing the percent identity and directly below the percent similarity for each species GST compared to *D. melanogaster*'s GST template. Figure S34. Genomic organization of the GSTs of the 11 *Drosophila* species. The genomic maps show the overall synteny of the GST gene family in the *Drosophila* genus. The genes are shown as arrows which indicate the direction of transcription. The blue color shows genes that FlyBase reports to have GST molecular function but does not specify a *D. melanogaster* ortholog. The magenta color shows pseudogenes identified by our curation. The purple color shows identified genes needing confirmation due to incomplete genome sequence. Figure S35. Phylogenetic tree analysis The phylogenetic tree analysis used sequences from each GST class from the 12 *Drosophila* species. Each isoform is designated by their FlyBase symbol followed by the *D. melanogaster* ortholog name in parenthesis. For example, Dana\GF17052(D1), where D1 refers to the DmelGSTD1 ortholog. In some species there is more than one isoform orthologous to the same D. melanogaster ortholog, so these will be noted as D1-2, D1-3 and so on. GSTT3A/B of *D. sechellia* and GSTT4 of *D. persimilis* were not included in this analysis due to incomplete gene sequencing. Table S1. Glutathione transferase orthologs in the 11 Drosophila species. Table S2. Example of alternative splicing curation of non-annotated genes for *D. yakuba*.
(RAR)
